# An fMRI dataset for whole-body somatotopic mapping in humans

**DOI:** 10.1038/s41597-022-01644-4

**Published:** 2022-08-23

**Authors:** Sai Ma, Taicheng Huang, Yukun Qu, Xiayu Chen, Yajie Zhang, Zonglei Zhen

**Affiliations:** 1 Beijing Key Laboratory of Applied Experimental Psychology, Faculty of Psychology, Beijing Normal University, Beijing, 100875 China; 2 State Key Laboratory of Cognitive Neuroscience and Learning & IDG/McGovern Institute for Brain Research, Beijing Normal University, Beijing, 100875 China

**Keywords:** Motor cortex, Cortex, Cognitive neuroscience

## Abstract

The somatotopic representation of the body is a well-established organizational principle in the human brain. Classic invasive direct electrical stimulation for somatotopic mapping cannot be used to map the whole-body topographical representation of healthy individuals. Functional magnetic resonance imaging (fMRI) has become an indispensable tool for the noninvasive investigation of somatotopic organization of the human brain using voluntary movement tasks. Unfortunately, body movements during fMRI scanning often cause large head motion artifacts. Consequently, there remains a lack of publicly accessible fMRI datasets for whole-body somatotopic mapping. Here, we present public high-resolution fMRI data to map the somatotopic organization based on motor movements in a large cohort of healthy adults (N = 62). In contrast to previous studies that were mostly designed to distinguish few body representations, most body parts are considered, including toe, ankle, leg, finger, wrist, forearm, upper arm, jaw, lip, tongue, and eyes. Moreover, the fMRI data are denoised by combining spatial independent component analysis with manual identification to clean artifacts from head motion associated with body movements.

## Abstract (summary)

**Table:** 

Measurement(s)	Functional brain measurement
Technology Type(s)	Blood Oxygen Level-Dependent Functional MRI
Factor Type(s)	Motor movements
Sample Characteristic - Organism	Homo sapiens
Sample Characteristic - Environment	Anthropogenic environment
Sample Characteristic - Location	China

## Background & Summary

Somatic representation of the body is a well-established organizational principle in the human sensorimotor cortex. In particular, body movement is mapped onto the primary motor and somatosensory cortices following the topographic relations of the body parts at a fine scale, known as the motor homunculus^1–3^. Similar somatotopic mapping is also found in a coarse manner in other regions of the motor system, including the supplementary motor area^4–6^, premotor cortex^7,8^, and cerebellum^9,10^. This topographical organization implements an internal body representation that is fundamental to precise motor control, spatial cognition, and social interaction^11^.

Direct electrical stimulation (DES) is the classic and main technique used to map the somatotopic organization of the cortex^1,5^. It achieves this by examining which parts of the body show positive movement effects (involuntary movements or twitches of muscles), while direct current is delivered to a site of the cortex in patients undergoing craniotomy. Although DES somatotopic mapping provides valuable insights into somatotopic organization, this procedure has two limitations^12,13^. First, DES can only be performed in eligible patients during surgery. This excludes it from being used to characterize individual variability in a large cohort of healthy participants. Second, DES can miss negative motor areas whose activities are inhibited by the applied stimulation^14^. Modern functional magnetic resonance imaging (fMRI) provides a complementary approach to DES to noninvasively map brain functions. Functional MRI has shown both high sensitivity and specificity for revealing somatotopic maps based on the voluntary movement task^3,9,15–21^. Unfortunately, body movements during acquisition of fMRI data often cause large head motion, which in turn can lead to severe artifacts in somatotopic mapping^22,23^. The recent publicly released Human Connectome Project(HCP) dataset contains a set of motor task fMRI data^24^. The somatotopic organization of the sensorimotor cortex has been revealed with the dataset though only finger, toe, and tongue movements were explicitly mapped in its motor task paradigm^25^. Consequently, there remains a lack of publicly accessible fMRI datasets for whole-body somatotopic mapping.

In this study, we present a public high-resolution fMRI data to characterize somatotopic maps *in vivo* in a large cohort of healthy adults (N = 62). The data are markedly different from existing fMRI data for somatotopic mapping. First, in contrast to previous studies that were mostly designed to distinguish body representations, such as the foot, hand, and tongue, almost all body parts that are allowed to move in the scanner were mapped in this study, including the toe, ankle, leg, finger, wrist, forearm, upper arm, jaw, lip, tongue, and eyes. Second, the data covered the whole brain, including the entire cerebellum, with unprecedented spatial resolution (2 mm isotropic) acquired on a Siemens 3T MAGNETOM Prisma scanner. Third, in contrast to previous studies with small sample sizes (N < 20), a large sample size was used (N = 62). This makes it possible to explore the interindividual variability of somatotopic maps in a healthy population. Finally, to remove the confounding artifacts caused by body movements and other factors, the fMRI data were denoised by combining spatial independent component analysis (ICA) with manual identification of artifacts^26^. Both head motion and physiological artifacts, including cardiac and respiratory noise, were effectively alleviated by this denoising procedure.

## Methods

### Participants

The study was approved by the Institutional Review Board (IRB) of Beijing Normal University. Flyers approved by the IRB were posted on campus network to recruit potential participants. To eliminate the possible confounding from handedness, right handedness was set as an inclusion criterion. Initially 68 right handed participants, who were screened using the revised Edinburgh Handedness Inventory^27^, were admitted into the experiment. All participants had normal or corrected-to-normal vision, reported no history of psychiatric or neurological disorders, and provided written informed consent prior to their participation. Six participants were excluded from somatotopic mapping fMRI experiment because they showed inferior performance in the behavior training (see section Behavior training for body movements). As a result, a total of 62 healthy participants (34 females), ranging in age between 19 and 29 years (mean ± standard deviation[SD], 22.76 ± 2.22 years), participated in the whole-body somatotopic mapping fMRI experiment. All participants provided their informed consent for sharing the anonymized data. The detailed demographic data of the participants are provided in Supplementary Table 1.

### Experimental procedure

To establish the topographic map of body movements in humans as comprehensively as possible, participants were instructed to perform movements of various body parts, including the toe, ankle, leg, finger, wrist, forearm, upper arm, jaw, lip, tongue, and eyes. The same movement was performed on both sides of the body at the same time, except for the legs. All movements were self-paced at a speed between 0.5 Hz and 5 Hz. The experimental conditions and associated movement patterns (i.e., instructions) for each condition are listed in Table 1. The experiment consisted of two behavior training sessions and one MRI scan session.

**Table 1:** The experimental conditions and associated movement patterns for each condition (i.e., body part).

Experimental conditions	Movement patterns for each condition
Toe movements	Flex and extend the toes from both feet.
Ankle movements	Dorsiflex and release both ankles.
Left leg movements	Lift and lower the left leg (maximum 10°) with the leg, ankle, and toes straight.
Right leg movements	Lift and lower the right leg (maximum 10°) with the leg, ankle, and toes straight.
Finger movements	Clench and loose both fists.
Wrist movements	Pitch and roll both wrists with clenched fists.
Forearm movements	Flex and release both forearms at the elbow (maximum 20°) with the wrists and fingers straight.
Upper arm movements	Lift and lower the upper arms (maximum 20°) with the upper arms, forearms, wrist, and fingers straight.
Jaw movements	Bite or twist jaws.
Lip movements	Expand and contract the lips with the teeth being bitten and tongue still.
Tongue movements	Circular tongue with the teeth being bitten and lips closed.
Eye movements	Blink or saccade eyes.
Rest	Fixate on the dot presented in the center of the screen.

#### Behavior training for body movements

Two behavior training sessions were completed before each participant was scanned to familiarize himself/herself with different types of movements and to reduce the possible head motion caused by the movements. First, the day before the experiment, the participants were instructed to lie supine on a comfortable bed to practice each movement following a video tutorial. The goal of the training was to have the participants master the required movement patterns, magnitude, and speed while avoiding head movements or movements from unrelated body parts. Second, prior to the MRI scan on the day of the experiment, the participants lying supine on a yoga mat were trained under the same visual instructions as those used in the fMRI scan. Feedback was provided by the experimenter to help participants refine their movements. Only participants whose performances were refined and qualified, as verified by two experimenters, were allowed to take part in the fMRI scan. Six participants were excluded because they showed inferior training performance.

#### Task paradigm for somatotopic mapping

**Fig. 1:**
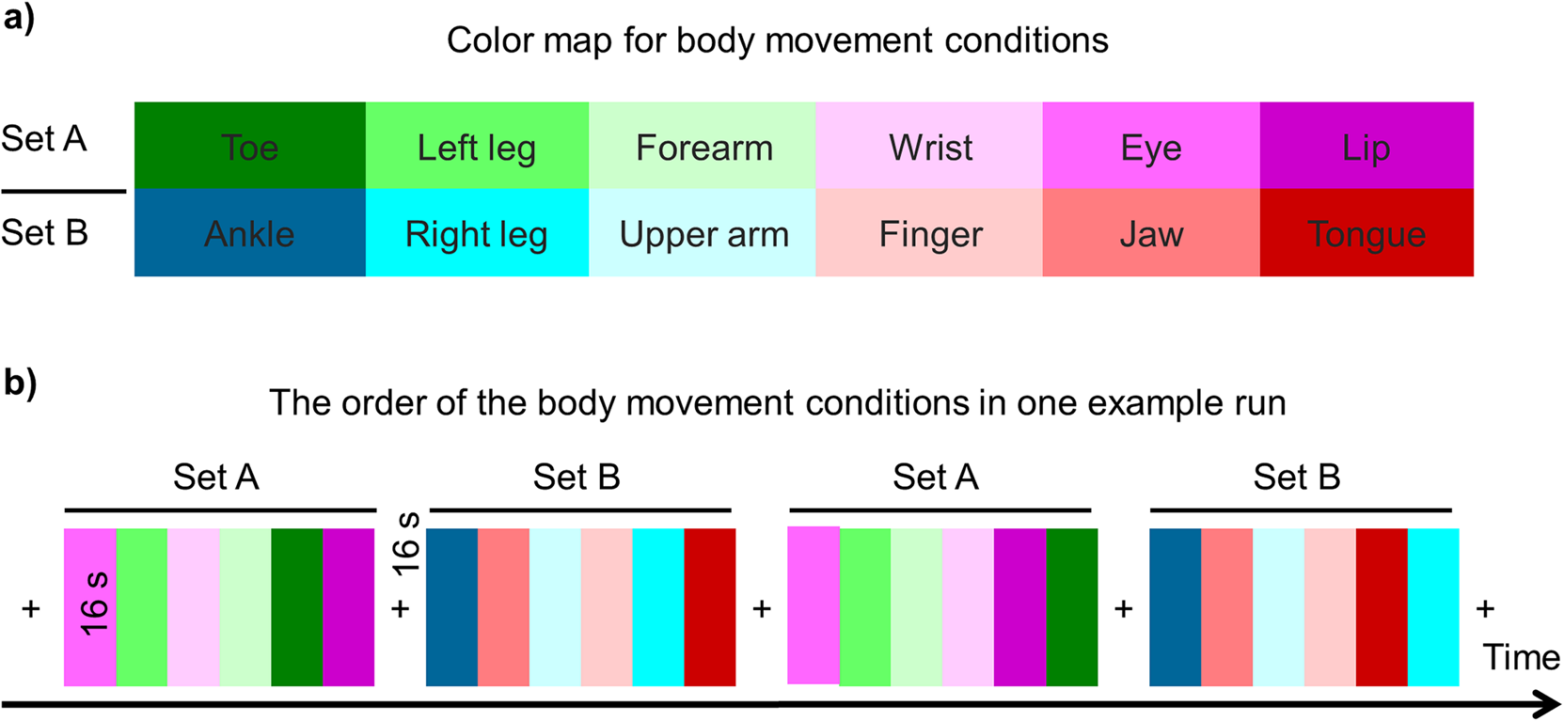
The blocked-design body movement task for mapping the topographical representations of human body. (**a**) The 12 body parts (i.e., conditions) were grouped into two sets to make the adjacent body parts into different groups as possible. (**b**) Each set was repeated twice in a run. The order of the set was counterbalanced, and the order of six movement conditions within each set was randomized.

Each participant underwent one fMRI session consisting of six blocked-design runs, each of which lasted 7 min 44 s. The experimental conditions consisted of the movements for each of twelve body parts, including toe, ankle, left leg, right leg, finger, wrist, forearm, upper arm, jaw, lip, tongue, and eye. Because the cortical motor and sensory systems are topographically organized (i.e., neighboring body parts are represented by neighboring brain areas), we grouped twelve different conditions (i.e., body parts) into two sets to make the conditions for adjacent body parts (e.g., toe and ankle) into distinct sets and thus reduce the overlap of BOLD signals from adjacent body parts. One set consisted of toe, left leg, forearm, wrist, eye, and lip, whereas the other consisted of ankle, right leg, upper arm, finger, jaw, and tongue (Fig. 1a). Each set was repeated twice, counterbalanced within a run, and intermixed with five rest blocks at the beginning, middle, and end of the run. The order of the conditions was randomized within each set and run. Both the movement and rest blocks lasted 16 s (Fig. 1b). In each movement condition, the participants maintained eye fixation and moved one of their body parts following the instructions presented on the screen. The instructions specifically indicated the moving part, moving pattern, and limits of the moving magnitude and speed (Table 1). During the rest period, the participant performed no movement while maintaining eye fixation. Notably, to acquire more data for movement conditions and thus increase the detection power of brain activations as much as possible, the rest blocks were only inserted between pair of condition sets instead of each pair of conditions. This strategy has been widely used in fMRI localizer for other domains such as visual object representations^28,29^. Moreover, it should be pointed out that no pauses inserted between movement conditions may cause a small delay between the instructional cue and the movement onset. However, as the blocked design is relatively insensitive to the small shift of the onset, we expected the small delay could be negligible in a block lasting 16 s.

The movement instructions were presented at the center of the screen within a visual angle of 4°, in a black monospaced font, on a gray background using the Psychophysics Toolbox Version 3(PTB-3)^30^ via an MR-compatible LCD screen. The vertical refresh rate of the LCD projector was 60 Hz. The head motion of the participants was restrained using a foam pillow and extendable padded head clamps. Moreover, head motion was monitored using a real-time video recording system. It was evaluated by the experimenters and fed back to the participants to help them improve their movements in the next run.

### Magnetic resonance imaging acquisition

Magnetic Resonance Imaging (MRI) was performed on a Siemens 3 Tesla (3 T) MAGNETOM Prisma MRI scanner at the BNU Imaging Center for Brain Research, Beijing, China, using a 64-channel phased-array head coil. We collected task-based fMRI, field map, and structural MRI in one scan session approximately 60 min duration. Six functional runs (7 min 44 s/run) for the body movement task were acquired in order. A spin-echo field map was acquired to correct the magnetic field distortion (2 min 27 s) between the third and fourth functional runs. A T1-weighted (T1w) anatomical image was obtained at the end (6 min). Earplugs were used to attenuate the scanner noise. The physiological data including heartbeat and breathing rates were not recorded. A summary of the session organization and MRI acquisition parameters can be found in Supplementary Table 2.

#### Functional MRI

Blood-oxygenation-level-dependent (BOLD) fMRI data were collected using a gradient-echo, multi-band (MB) accelerated echo-planar imaging T2*-weighted sequence with the following parameters: 72 transversal slices parallel to the AC-PC line; in-plane resolution, 2 × 2 mm; slice thickness, 2 mm without gap; phase encoding, A» P; field of view (FOV), 200 × 200 mm; repetition time (TR), 2000 ms; echo time (TE), 34.0 ms; flip angle, 90°; echo spacing, 0.54 ms; bandwidth, 2380 Hz/Px; and MB factor, 3.

#### Field map

The two-dimensional spin-echo maps were acquired using the following parameters: 72 slices with the same position as fMRI; slice thickness, 2 mm without gap; voxel size, 2 × 2 × 2 mm; phase encoding, R» L; FOV, 200 × 200 mm; TR, 720 ms; TE1/TE2, 4.92/7.38 ms; and flip angle, 60°.

#### Structural MRI

Structural T1w images were acquired with a three-dimensional magnetization-prepared rapid acquisition gradient echo sequence: 1 slab; 208 sagittal slices; FOV, 256 × 256 mm; slice thickness, 1 mm; isotropic voxel size, 1 × 1 × 1 mm; phase encoding, A» P; TR, 2530 ms; TE 2.27 ms; TI, 1100 ms; and flip angle, 7°.

## Data Analysis

### Data organization

The Digital Imaging and Communications in Medicine (DICOM) images acquired from the Siemens scanner were converted into the Neuroimaging Informatics Technology Initiative (NIfTI) format and then reorganized into the Brain Imaging Data Structure (BIDS) using HeuDiConv (https://github.com/nipy/heudiconv). The anatomical T1w images were anonymized by removing facial features using the PyDeface (https://github.com/poldracklab/pydeface).

### Data preprocessing

The data were preprocessed using fMRIPrep 20.2.1^31^. Each T1w volume was corrected for intensity nonuniformity using N4BiasFieldCorrection^32^ and skull-stripped using antsBrainExtraction^33^. The brain surfaces were reconstructed using recon-all from FreeSurfer^34^. Spatial normalization to the ICBM 152 Nonlinear Asymmetrical template version 2009c was performed using antsRegistration^33^. Brain tissue segmentation was performed on the brain-extracted T1w images using FAST^35^. Functional data were motion-corrected using MCFLIRT^36^. SDCflows was used to estimate the field map on the phase-difference B0 maps and to correct the field distortion of the fMRI data (https://www.nipreps.org/sdcflows). This was followed by boundary-based co-registration to the corresponding T1w using bbregister^37^. Finally, motion-correcting transformations, BOLD-to-T1w transformation, and T1w-to-template (MNI) warp were concatenated and applied in a single step using antsApplyTransforms with Lanczos interpolation^33^. No additional spatial or temporal filtering was applied. Further details on the fMRIPrep pipeline can be found in fMRIPrep documentation (https://fmriprep.org).

### Data denoising

To reduce confounding artifacts from head motion and physiological factors (e.g., cardiac and respiratory), the fMRI dataset was denoised separately for each run from each participant (i.e., 6 × 62 = 372 runs in total) by manually identifying artifacts from the spatial ICA. Specifically, a spatial ICA was performed on each run from each participant in the individual native space using MELODIC (version 3.15) from the FSL with default parameters^38^. Nine categories of artifact-related ICs (A-ICs) and two categories of signal ICs (S-ICs) were then manually identified according to their spatial maps, time courses, and power spectrum of the time courses using melview (https://fsl.fmrib.ox.ac.uk/fsl/fslwiki/Melview)^26,39^.The A-ICs included artifacts from head motion, susceptibility motion, non-brain, respiratory, cardiac, sagittal sinus, white matter, MRI hardware, and unclear sources. The S-ICs included known and unknown signals. A known signal is a well-known characteristic of neural-related signals and is correlated with some of the body movement waveforms. The unknown signal shows neither typical characteristics of neural-related signals nor clear characteristics of artifacts. The ICs were labeled as unknown signals to preserve as much of the signal of interest as possible. The characteristics of the different IC categories are summarized in Table 2. Examples of labeled S-ICs and A-ICs are provided in Supplementary Figs. 1–11. Overall, 101 ± 13 (mean ± SD) ICs were decomposed from one run of the fMRI data; on average, 43 ± 10 and 58 ± 11 (mean ± SD) of them were labeled as S-ICs and A-ICs, respectively. After the A-ICs were identified for each run, the data were cleaned using partial regression of the time series of the A-ICs from the original data.

**Table 2:** Spatial, temporal, and power characteristics of different categories of ICs.

Category	Spatial map	Time series	Power spectrum
Signal	Small number of contiguous clusters of voxels in gray matter	Correlated with some movement conditions	Predominantly in low frequencies (<0.1 Hz)
Head motion	Ring-like shape or stripes around the edge of the brain	Sudden jumps or gradual drifts (correlated with realignment parameters)	Broadband, but dominated by low-frequency content
Susceptibility motion	Areas near air cavities or blood vessels	Sudden jumps and correlated with realignment parameters	Predominantly in low frequencies (<0.1 Hz)
Non-brain	Eyeball, tongue, and throat	Regular oscillatory patterns	Predominantly in low frequencies (<0.1 Hz)
Cardiac	Contiguous clusters of voxels overlapped with the known anatomical structures	Regular oscillatory patterns, no sudden jumps or gradual change	Usually dominated by high frequencies (>0.1 Hz), sometimes aliased into low frequencies (<0.1 Hz)
Respiratory			
Sagittal sinus			
White matter			
MRI related	Abrupt intensity changes in slice direction	Sudden jumps and/or oscillation patterns	Predominantly in high frequencies (>0.1 Hz)
Unclassified noise	Mixture of multiple types of artifacts	Sudden jumps and/or oscillation patterns	Broadband
Unknown signal	Usually a mixture of signal and noise, hard to be identified as signal or noise unambiguously.		

### Activation analysis

The manually denoised data were adapted to a CIFTI-based grayordinate format using Ciftify to allow combined cortical surface and subcortical volume analyses^40^. Specifically, anatomical data were converted from FreeSurfer to CIFTI formats. MNI inter-subject anatomy-based registration and resampling were performed using the ciftify_recon_all function. Functional MRI data were projected onto the surface using the cifitfy_subject_fmri function. Statistical analyses of the time series were performed using the TaskfMRIAnalysis script from Human Connectome Project (HCP) pipelines^41^, which ran FMRIB’s improved linear model with a local autocorrelation correction for each run^42^. A predictor was created for each of the twelve movement conditions by convolving the boxcar function of each condition with a gamma hemodynamic response function. The temporal derivative was also added to the model. Because the data had been denoised manually, no nuisance variables were included in the linear model. A fixed-effects model was used to summarize the parameter (i.e., beta) images across runs for each participant. Finally, the group-level analysis was performed for each contrast of interest using a one-sample t-test with beta images from all participants as inputs via the TaskfMRIAnalysis script from HCP pipelines, which incorporates FLAME (FSL’s Local Analysis of Mixed Effects) to provide accurate parameter estimates^43^.

## Data Records

The data were organized according to the Brain-Imaging-Data-Structure (BIDS) Specification version 1.7.0^44^ and can be accessed from the OpenNeuro public repository (accession number: ds004044)^45^. All MRI data were successfully collected from each participant. No special circumstances occurred in data collection and no anomalies were found in further data processing.

### Raw data

Structural MRI: <Sub-ID>/ses-1/anat/<SUB-ID>_ses-1_run-01_T1w.nii.gz

Functional MRI: <Sub-ID>/ses-1/func/<Sub-ID>_ses-1_task-motor_<Run-ID>_bold.nii.gz

Field mapping: <Sub-ID>/ses-1/fmap/<Sub-ID>_ses-1_run-01_<magnitude/phasediff>.nii.gz

Task events: <Sub-ID>/ses-1/func/<Sub-ID>_ses-1_task-motor_<Run-ID>_events.tsv. The event tabular file describes onset, duration and trial type (i.e., condition) for each block during a run.

### Preprocessed data

Preprocessed functional MRI: derivatives/fmriprep/<Sub-ID>/<Sub-ID>_ses-1_task-motor_<Run-ID>_space-T1w_desc-preproc_bold.nii.gz

### Manually denoised fMRI data

Denoised fMRI: derivatives/fmriprep/<Sub-ID>/<Sub-ID>_ses-1_task-motor_<Run-ID>_space-T1w_desc-preproc_bold_denoised.nii.gz

Spatial maps from ICA: derivatives/melodic/<Sub-ID>/ses-1/<Sub-ID>_ses-1_task-motor_<Run-ID>.ica/melodic_IC.nii.gz

Time series from ICA: derivatives/melodic/<Sub-ID>/ses-1/<Sub-ID>_ses-1_task-motor_<Run-ID>.ica/melodic_mix

Manually classified labels: derivatives/melodic/<Sub-ID>/ses-1/<Sub-ID>_ses-1_task-motor_<Run-ID>.ica/classified_labels.csv

### Brain activation data

Native surface: derivatives/ciftify/<Sub-ID>/native_surface

Results of task analysis: derivatives/ciftify/<Sub-ID>/GLM

## Technical Validation

The quality of the datasets was validated in four aspects. First, the brain coverage of the data was evaluated. Second, we assessed the magnitude of head motion. Third, we showed that the denoising procedure remarkably improved the quality of the data. Finally, we demonstrated the potential of the data in mapping fine-scale topographical representations of body movements.

### The data cover the whole brain

**Fig. 2:**
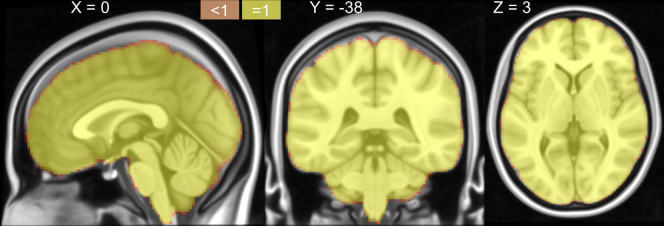
The coverage probabilistic map provided a voxelwise description for the brain coverage, indicating all brain structures were covered in each individual.

Previous studies have shown that self-paced body movements can induce extensive brain activation in cortex, subcortical and cerebellum. To check whether our data could completely cover these brain structures, a coverage probabilistic map was computed across all participants in the MNI space. The map coded the probability that each location would be recorded across the participants. As shown in Fig. 2, all of the locations within the MNI152 template are perfectly covered (i.e., 100% covered), indicating that the data are suitable for mapping fMRI responses to movements from any brain structure, including the subcortical cortex and cerebellum.

### The head motion is in good control

**Fig. 3:**
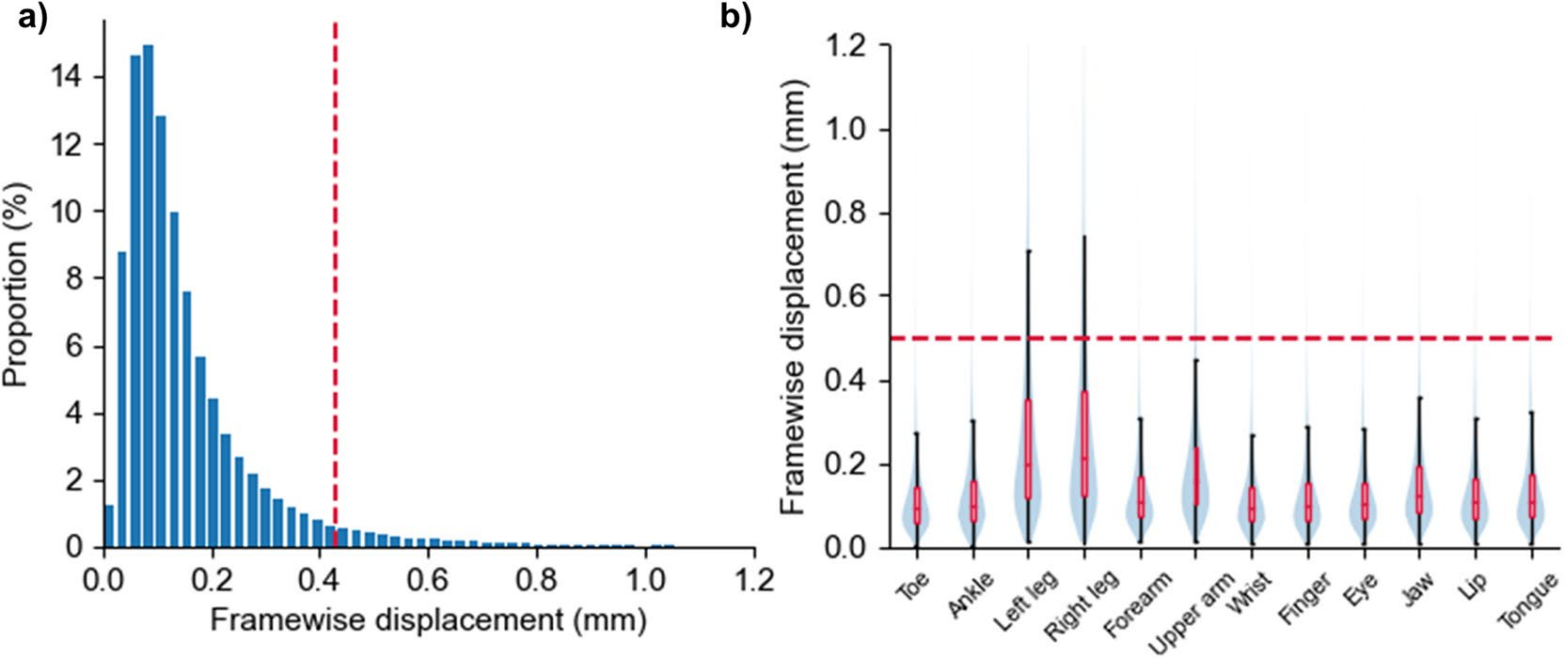
The distribution of head motion magnitude measured by framewise displacement (FD). (**a**) The histogram of FD calculated from all runs and participants. The long tail of the distribution indicates that instantaneous head motion is small. (**b**) The histogram of FD for different movement conditions displayed as violin plots.

Beyond all questions, head motion is the major confounding factor in acquiring fMRI data to establish a topographic map of body movements because the movements of body parts inevitably lead to head movements. The mean framewise displacement (FD), which measures instantaneous head motion by comparing the motion between the current and previous volumes^46^, was calculated to evaluate whether the magnitude of head motion is acceptable in the data. As shown in Fig. 3a, the 95% coverage interval of the FD from all volumes, runs, and participants appeares approximately within [0, 0.43], indicating that head motion is not unusual in performing body movements. As expected, head motion varied much across the movement conditions (Fig. 3b). Leg and upper arm movements caused larger head motions than the other conditions. However, even for these two conditions, only a few volumes show FD larger than 0.5 mm, which is often used as a criterion to identify the volume with large head motion in the literature^46^. These results indicate that head motion is in good control under our carefully designed tasks and experimental protocols.

### The data show high temporal signal-to-noise ratio

**Fig. 4:**
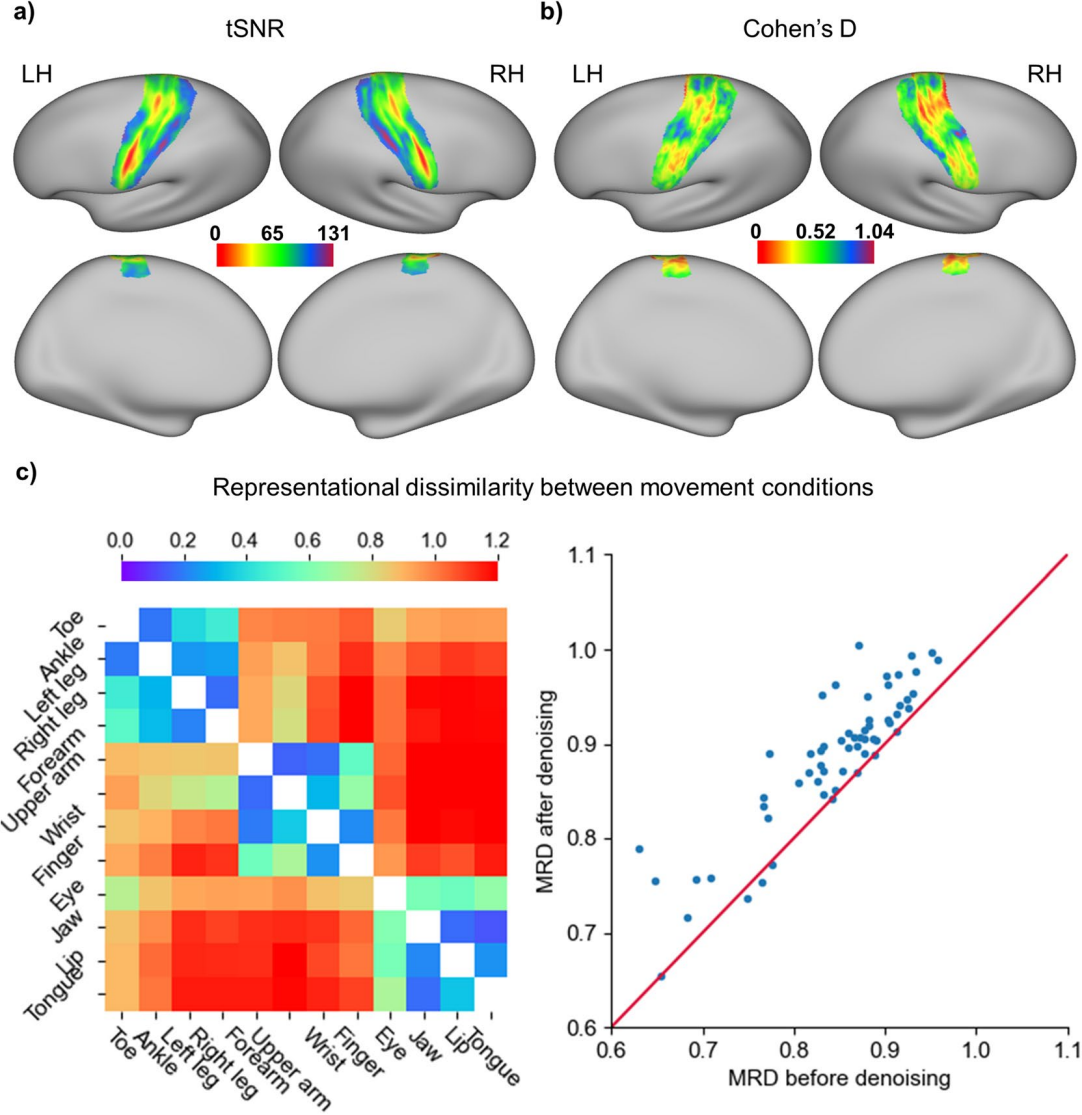
The functional magnetic resonance imaging (fMRI) data showed high temporal signal-to-noise ratio (tSNR) within somatotopic cortices. The tSNR was calculated for each vertex within the somatotopic region of interest on the fsLR surface and averaged across all runs and participants. (**a**) The tSNR map for the preprocessed fMRI data before independent component analysis (ICA)-based denoising. (**b**) Cohen’s d effect size of the tSNR improvement. Cohen’s d was calculated by dividing the mean difference by the standard deviation of the difference between the tSNR from the fMRI data before and after ICA-based denoising. (**c**) Left: The group-averaged representational dissimilarity matrix for the 12 body movement conditions, calculated from the data before (lower triangular part) and after (upper triangular part) ICA-based denoising. Right: The scatterplot of the mean representational dissimilarities (MRD) across all pairs of conditions calculated on each individual. Almost all of the participants show improved representational dissimilarities after the data were denoised.

Next, we characterized the signal-to-noise ratio (SNR) of fMRI time series in terms of temporal SNR (tSNR). The tSNR was computed on a vertex-wise basis on the surface and defined as the mean of each vertex’s time course divided by its standard deviation for each run. Because we were interested in the brain areas that are associated with movement and sensory representations of various body parts, we constrained these analyses within the somatotopic region of interest, which was generated by combining areas 1, 2, 3a, 3b,and 4 in the multimodal parcellation atlas^25^. More than 95% of the vertices show tSNR values greater than 50 on the preprocessed data without ICA-based denoising, indicating that the data are of good quality even before denoising (Fig. 4a). The ICA-based denoising procedure, in which the signal and noise components were manually identified, further improved the tSNR of the data. The Cohen d effect size of the tSNR improvement is larger than 0.5 for most vertexes, indicating a moderate to large effect of the denoising procedure (Fig. 4b). Importantly, the representational dissimilarities between different body parts, which were calculated as the correlation distance (1-r) between multi-voxel activity patterns from each pair of conditions, grow larger in the denoised data on both group averages and individuals (Fig. 4c), indicating that the neural representations for different body parts become more distinct after denoising. Altogether, these results demonstrate that the data are of good quality, particularly after ICA-based denoising.

### The data could reveal topographical representations of different body parts

**Fig. 5:**
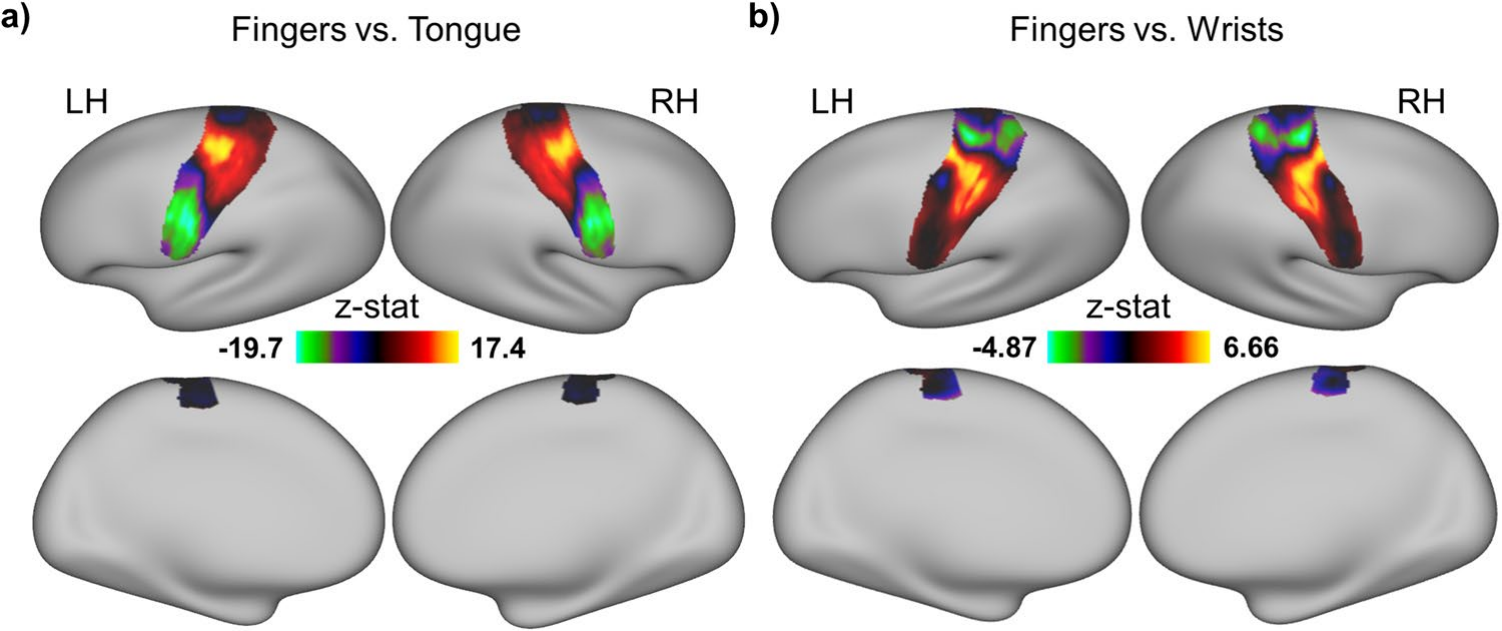
The activation maps from two example contrasts. The group-level analysis was performed for two contrasts of interest using a one-sample t-test with the beta images from all participants as inputs. The analysis was constrained within the primary somatotopic region. To reveal the continuous changes of topographical activations within the region, no threshold was used in displaying the activation maps. (a) Group-level z-statistical parametric maps for the contrast fingers vs. tongue. Red indicates fingers > tongue, whereas green indicates tongue > fingers. (b) Group-level z-statistical parametric maps for the contrast fingers vs. wrists. Red indicates fingers > wrists, whereas green indicates wrists > fingers.

To demonstrate the potential of the data in mapping brain representation for different body parts, brain activation maps from two example contrasts were examined: the fingers versus the tongue and the fingers versus the wrists. The former aimed to map the two body parts that are far apart (i.e., two brain areas located distantly), whereas the latter was used to map the two body parts that are close together (i.e., two brain areas located adjacently). As shown in Fig. 5a, the activations for both the fingers and tongue are well identified at the expected locations. The fingers are localized in the dorsal part of the motor and somatosensory cortices, whereas the tongue is localized in the ventral parts. Moreover, the wrists activations are well separated from the fingers’ activations. The wrists are localized more dorsally than the fingers although they are very close to each other (Fig. 5b). Together, these analyses demonstrate that the data have the potential to map the topographical representation of body movements in humans.

## Usage Notes

Here, we presented a public fMRI dataset for whole-body somatotopic mapping acquired from a large cohort of participants (N = 62) with a 2-mm isotropic spatial resolution. A manual denoising procedure was used to remove head motion artifacts caused by body movements during the task. These data provide unique opportunities to study the motor and somatosensory systems of the human brain at high resolution. First, the data are ideal for examining the topographical representation of body movements in humans (see Technical Validation). Precise mapping of individual motor representations and characterization of variabilities among individuals will lay the foundation for further understanding the function of different motor and somatosensory areas. Second, the data are suitable for constructing an *in vivo* brain atlas for motor areas in humans, which in turn can supply a quantitative spatial reference system to integrate multiple sources of information (e.g., postmortem histology or MRI) to understand the structure and function of human motor and somatosensory systems^47,48^. Finally, the data can be used to examine how different motor areas interact in body movements using approaches similar to resting state functional connectivity analysis^17,49,50^.

Although we believe that this dataset is a unique resource for mapping the topographical representation of body movements in humans, we should acknowledge its limitations. First, in our task paradigm, the adjacent body parts were arbitrarily divided into distinct sets to reduce the possible overlap of BOLD signal from conditions for adjacent body parts. Although this choice could increase the detection power for the brain activations from each body part, it results in that the dataset cannot be used to examine the interactions between neighboring body part representations because the areas for neighboring body parts were even not activated at the same time. Second, to save time in acquiring as many functional volumes as possible and thus improve the power of activation detection, we did not collect resting-state fMRI and diffusion MRI data. This makes it impossible to directly explore the association among task activation, resting-state functional connectivity, and anatomical connectivity in individuals. Third, only right-handed participants were included in this experiment. This limits the possibility of examining how finer-grained motor topology is affected by handedness. Finally, although the finger movements were mapped in the task, the data cannot afford to map individual fingers because subjects were asked to move all fingers simultaneously in the finger movement condition. It would be a good attempt to map individual fingers by combining our data with other publicly available fMRI data in which the representations of the individual fingers can be resolved^51,52^.

## Supplementary information

Supplementary Table 1

Supplementary Table 2

Supplementary Figures 1-11

## Data Availability

All codes for the experimental design, data organization, and technique validation are available at https://github.com/BNUCNL/WholebodySomatotopicMapping. Preprocessing was performed using fMRIPrep version 20.2.1 (https://fmriprep.org). ICA was performed using MELODIC version 3.15 (https://fsl.fmrib.ox.ac.uk/fsl/fslwiki/MELODIC), and IC classifications were manually performed using melview (https://fsl.fmrib.ox.ac.uk/fsl/fslwiki/Melview). Grayordinate-based (CIFTI format) brain activation analysis was performed by combining the Ciftify (https://github.com/edickie/ciftify) and HCP pipelines (https://github.com/Washington-University/HCPpipelines).
