# Supplementary material for: An fMRI dataset for whole-body somatotopic mapping in humans
Source: Sci Data. 2022 Aug 23;9:515. doi: 10.1038/s41597-022-01644-4 (PMC9399117; doi:10.1038/s41597-022-01644-4)
Supplement: Supplementary file 3 — Supplementary Figures 1-11 [file 41597_2022_1644_MOESM3_ESM.pdf]

Supplementary Figures

Supplementary Figure 1 2
Supplementary Figure 2 3
Supplementary Figure 3 4
Supplementary Figure 4 5
Supplementary Figure 5 6
Supplementary Figure 6 7
Supplementary Figure 7 8
Supplementary Figure 8 9
Supplementary Figure 9 10
Supplementary Figure 10 11
Supplementary Figure 11 12

Supplementary Figure 1. Signal

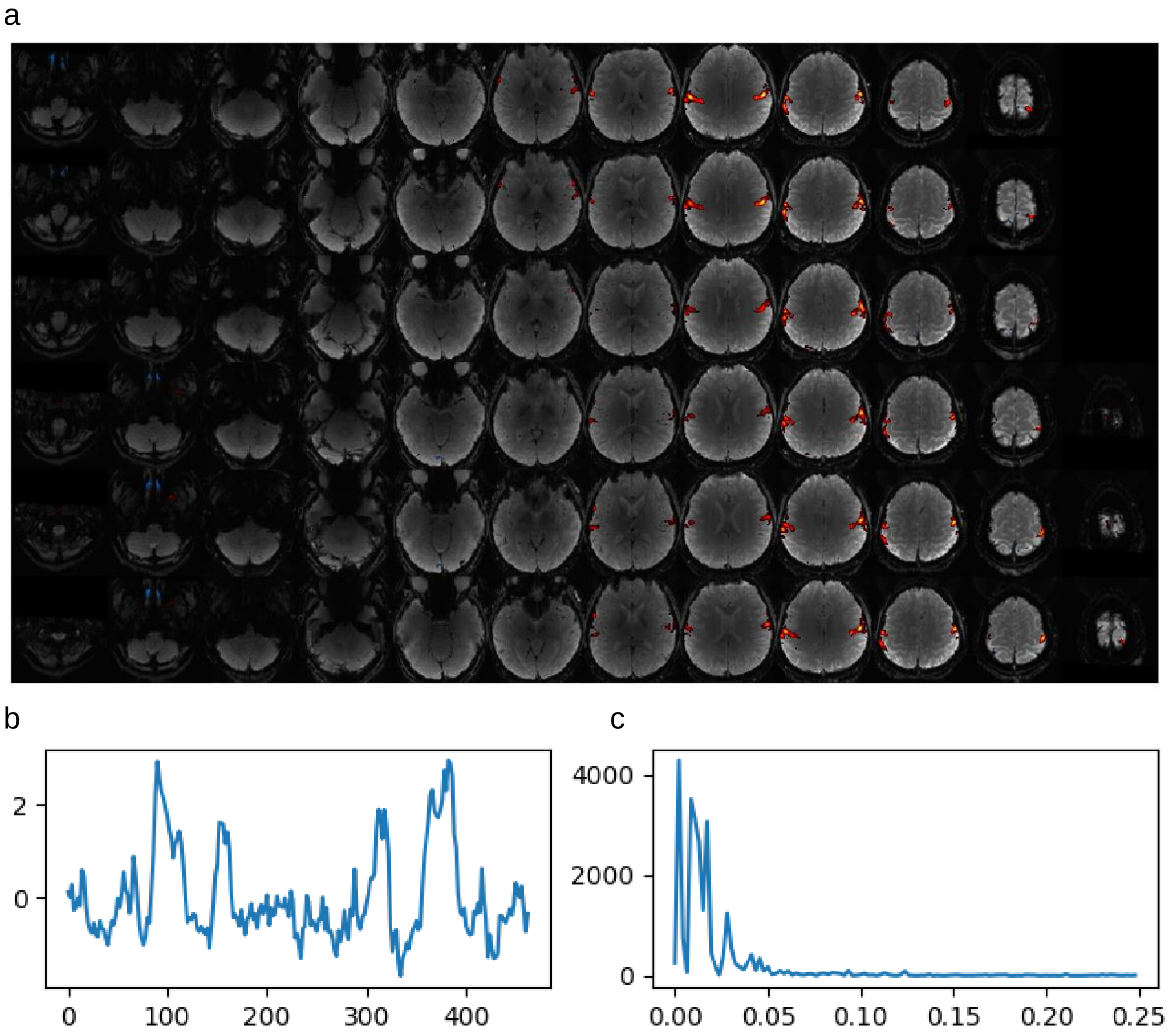

Supplementary Figure 1. S-IC example, Known signal (sub-18, run-1, IC-027). The spatial map of this category (thresholded at Z > 2.3, the following figures also applies this rule) showed small number of contiguous clusters of voxels in gray matter **(a)**. The time series of this category showed a task related waveforms, without sudden jumps **(b)**. The spectral power of this category lied predominantly in low frequencies (i.e., < 0.1Hz) **(c)**.

Supplementary Figure 2. Unknown signal

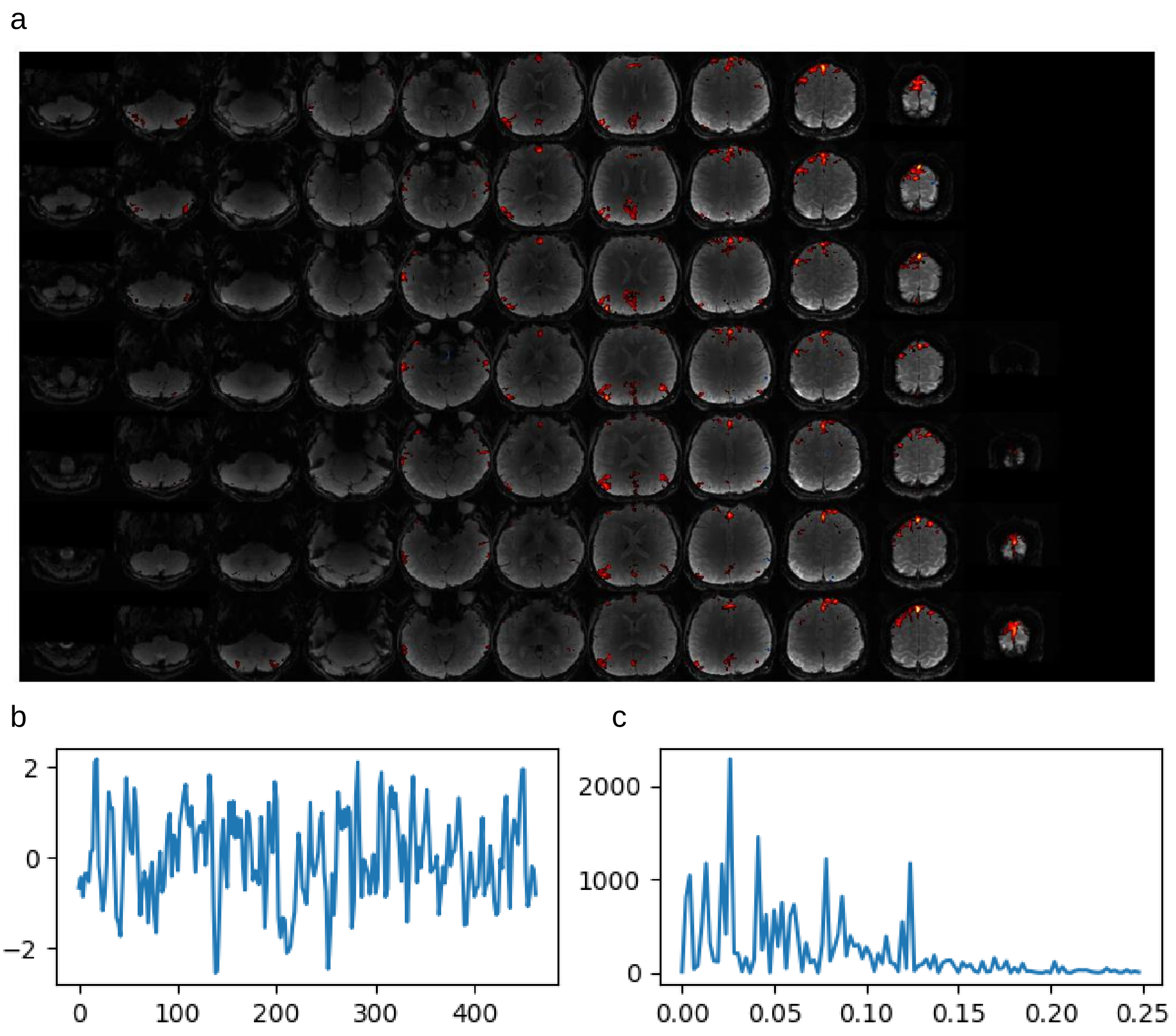

Supplementary Figure 2. S-IC example, Unknown signal (sub-20, run-3, IC-042). The spatial map of this category also showed small number of contiguous clusters of voxels in gray matter like S1 **(a)**. The time series of this category showed regular oscillatory patterns, without task related waveforms **(b)**. The spectral power of this category lied predominantly in low frequencies (i.e., < 0.1Hz) **(c)**.

Supplementary Figure 3. Head motion

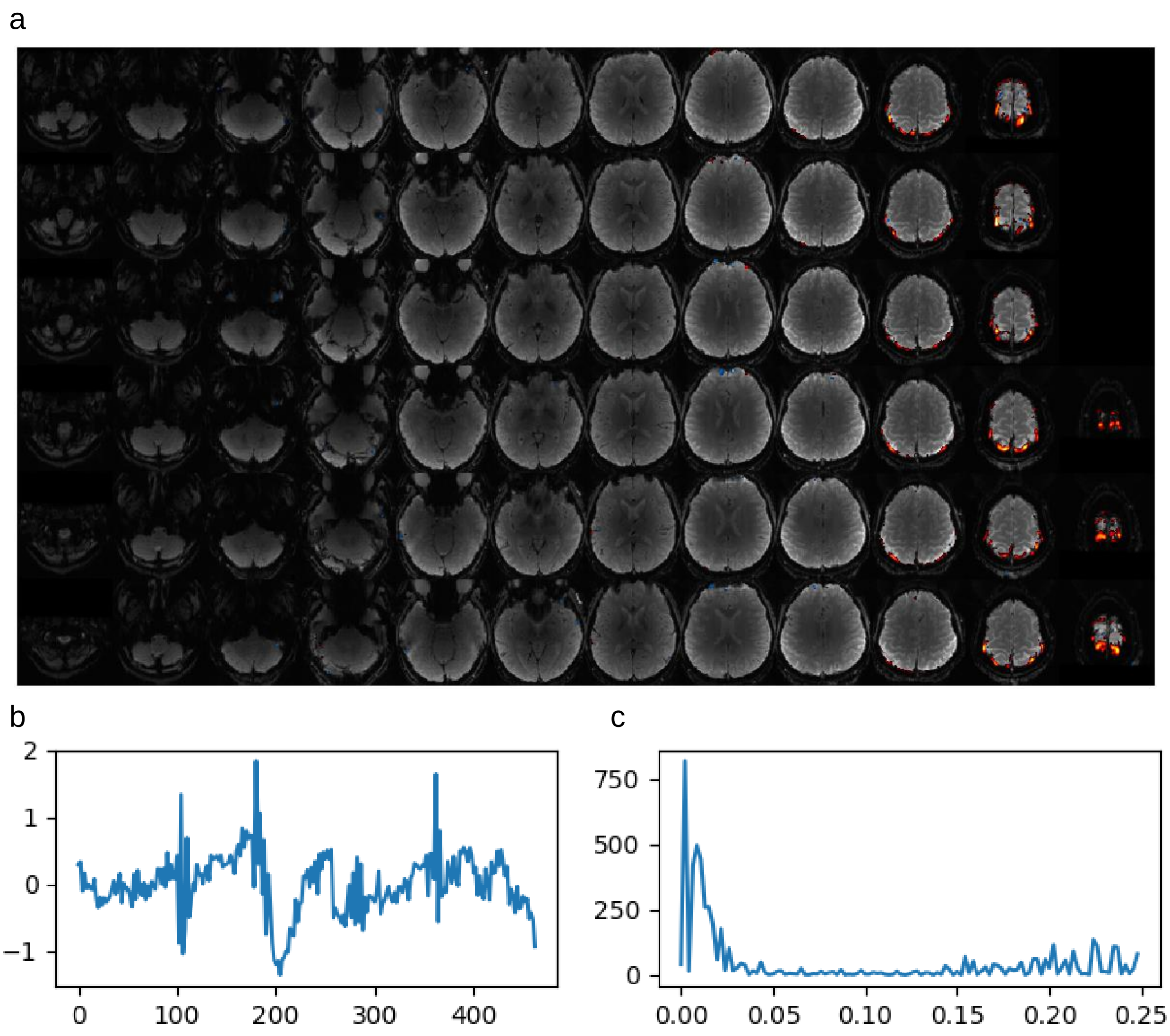

Supplementary Figure 3. A-IC example, Head motion (sub-18, run-4, IC-001). The spatial map of this category showed ring-like shape or stripes around the edge of the brain **(a)**. The time series of this category showed a realignment parameters related waveforms, with sudden jumps or gradual drifts **(b)**. The spectral power of this category lied predominantly in low frequencies (i.e., < 0.1Hz) **(c)**.

Supplementary Figure 4. Susceptibility-motion

a

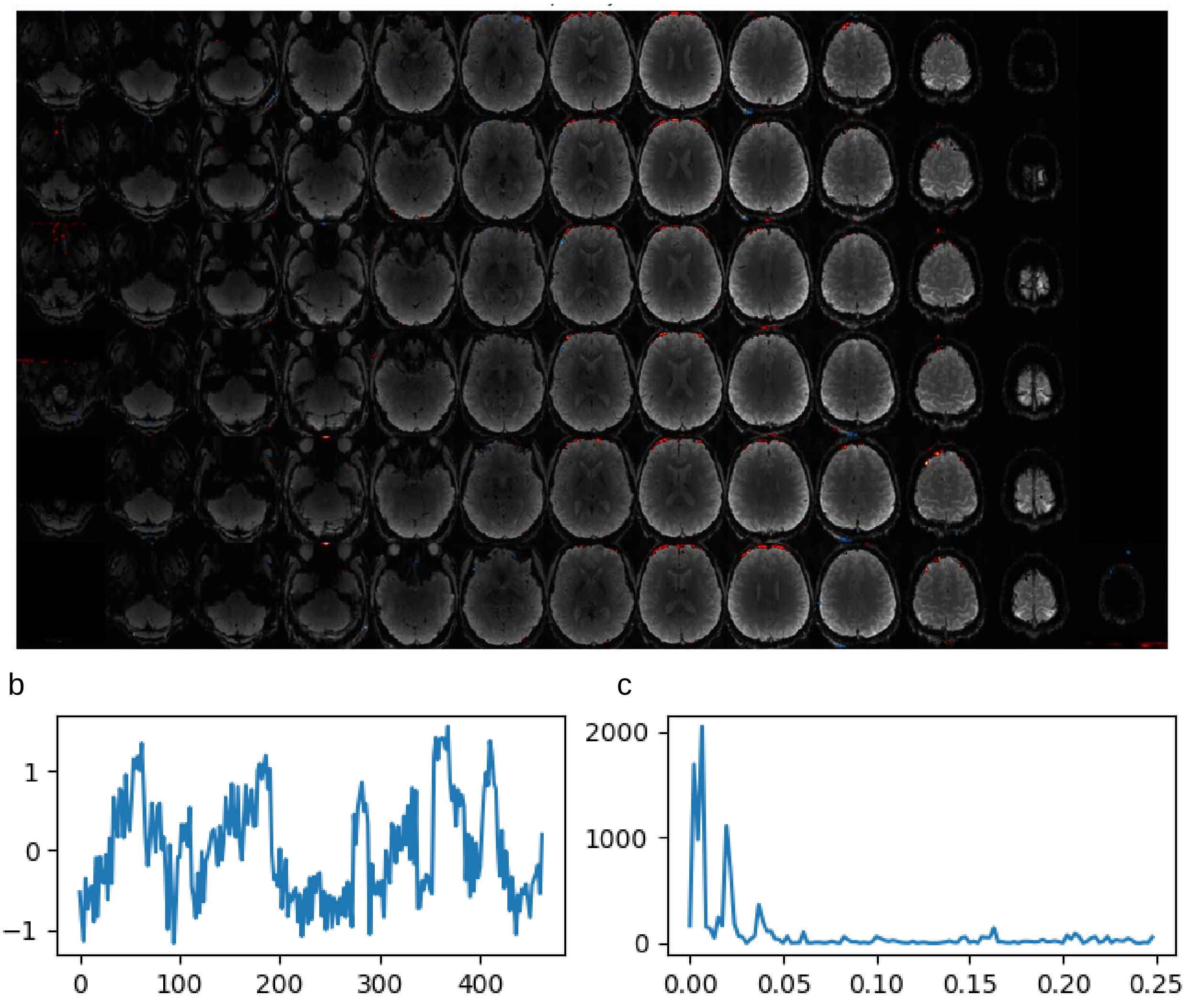

Supplementary Figure 4. A-IC example, Susceptibility-motion (sub-21, run-3, IC-002). The spatial map of this category showed near air cavities or blood vessels **(a)**. The time series of this category showed a realignment parameters related waveforms, with sudden jumps **(b)**. The spectral power of this category lied predominantly in low frequencies (i.e., < 0.1Hz) **(c)**.

Supplementary Figure 5. Non-brain

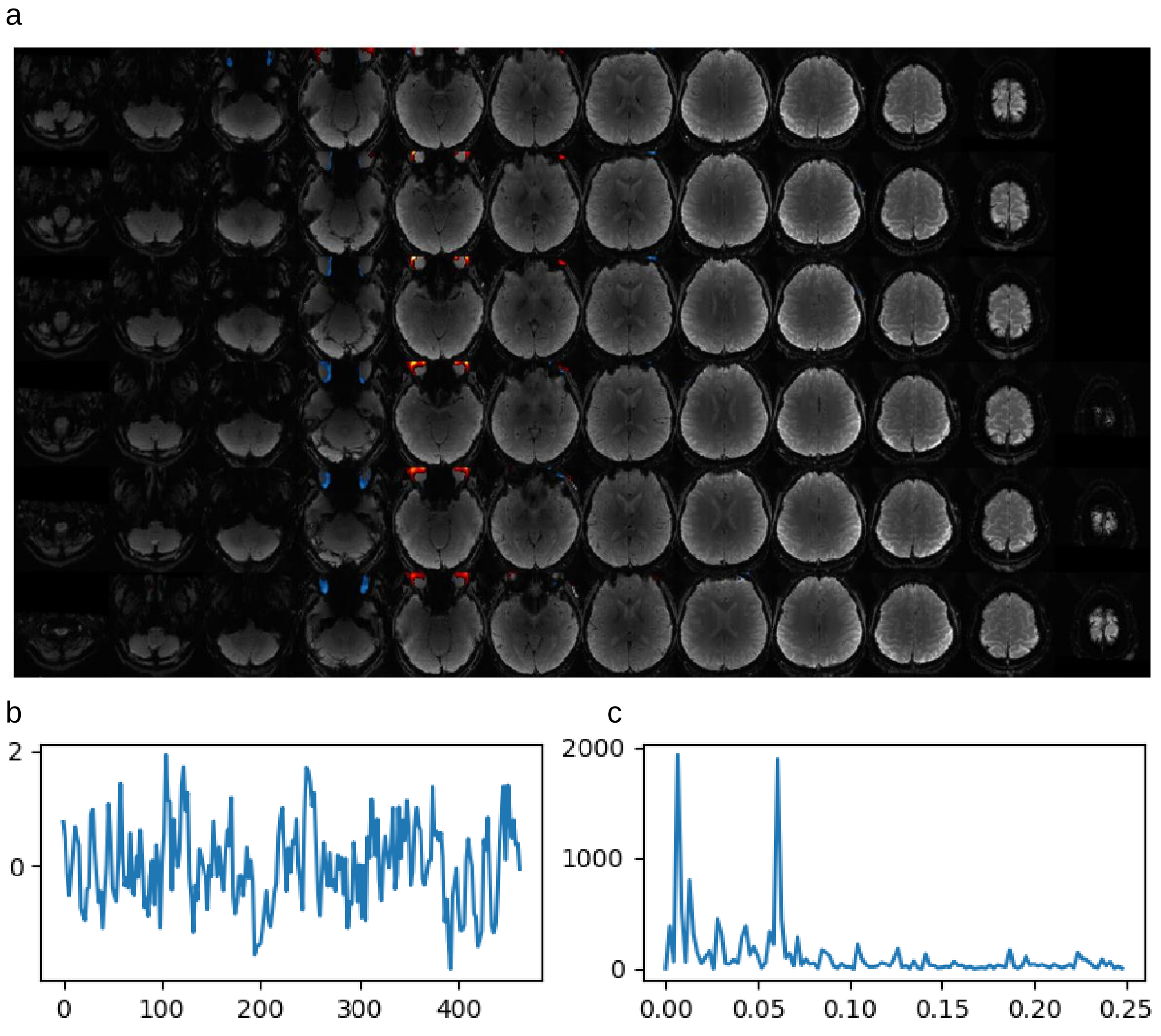

Supplementary Figure 5. A-IC example, Non-brain (sub-18, run-1, IC-016). The spatial map of this category showed in eyeball **(a)**. The time series of this category showed regular oscillatory patterns, without task related waveform **(b)**. The spectral power of this category lied predominantly in low frequencies (i.e., < 0.1Hz) **(c)**.

Supplementary Figure 6. Cardiac

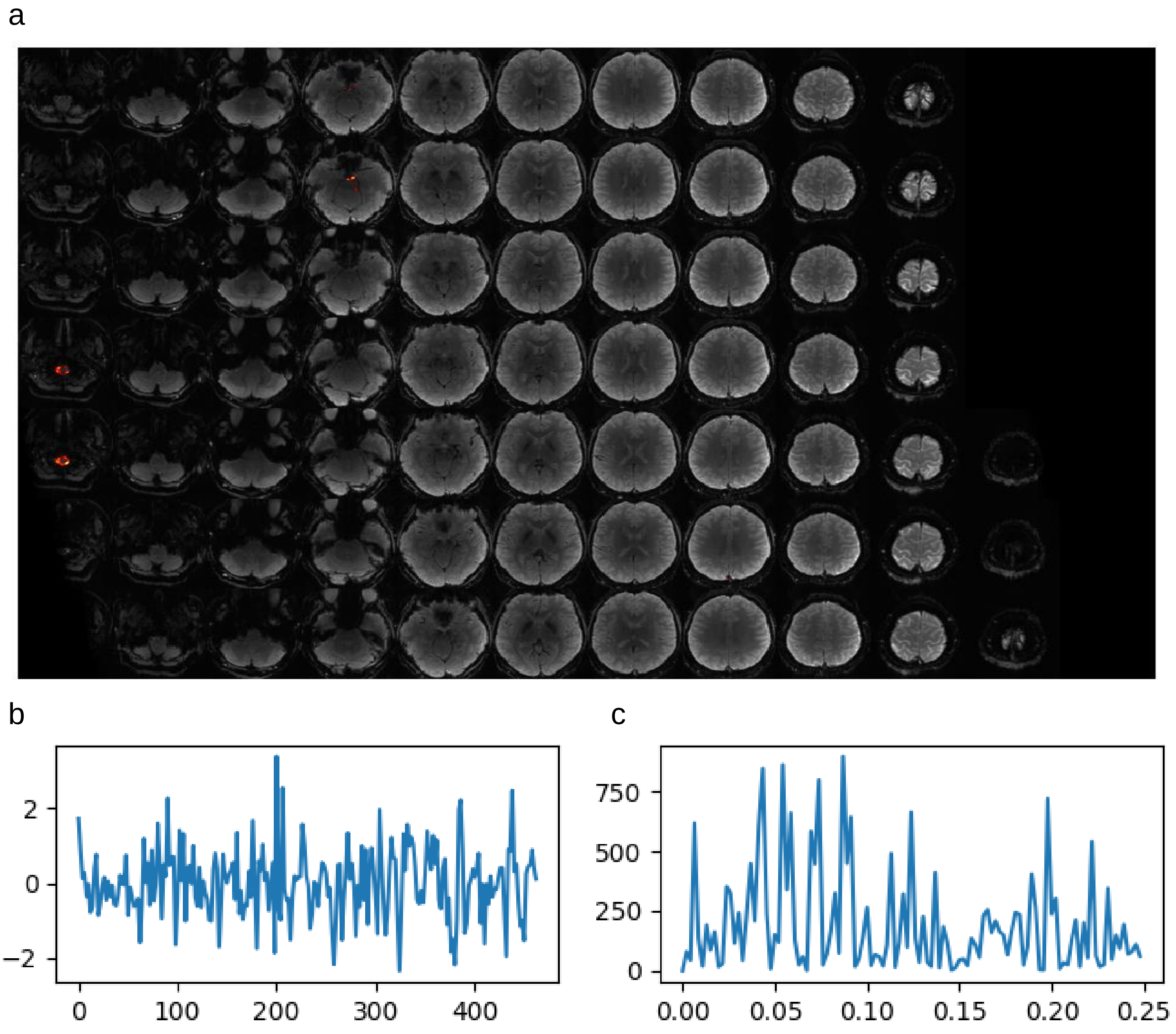

Supplementary Figure 6. A-IC example, Cardiac (sub-30, run-4, IC-071). The spatial map of this category showed in artery **(a)**. The time series of this category showed regular oscillatory patterns, without sudden jumps or gradual change **(b)**. The spectral power of this category lied in broad bands **(c)**.

Supplementary Figure 7. Respiratory

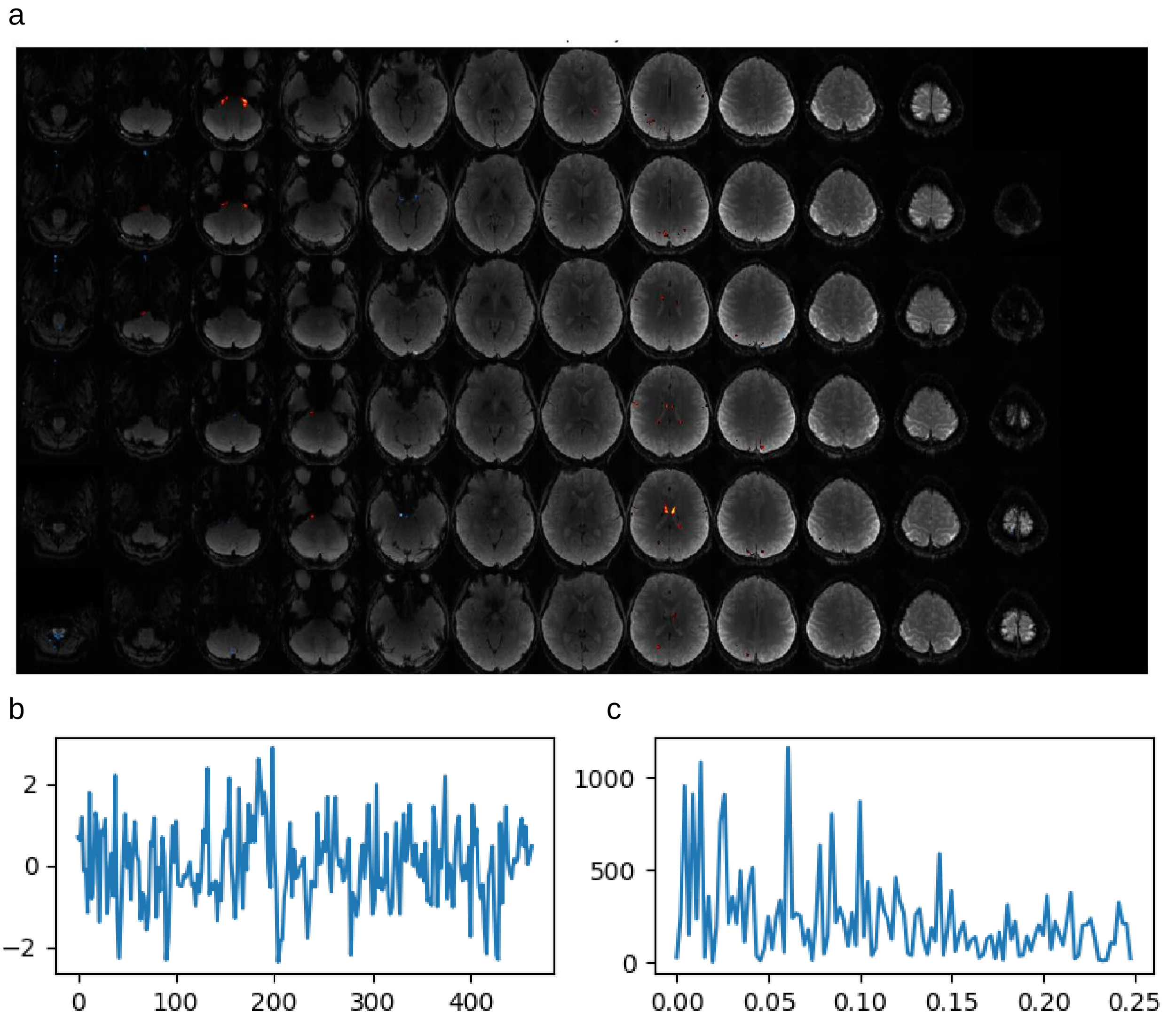

Supplementary Figure 7. A-IC example, Respiratory (sub-29, run-5, IC-091). The spatial map of this category showed in ventricles **(a)**. The time series of this category showed regular oscillatory patterns, without sudden jumps or gradual change **(b)**. The spectral power of this category lied in broad bands **(c)**.

Supplementary Figure 8. Sagittal sinus

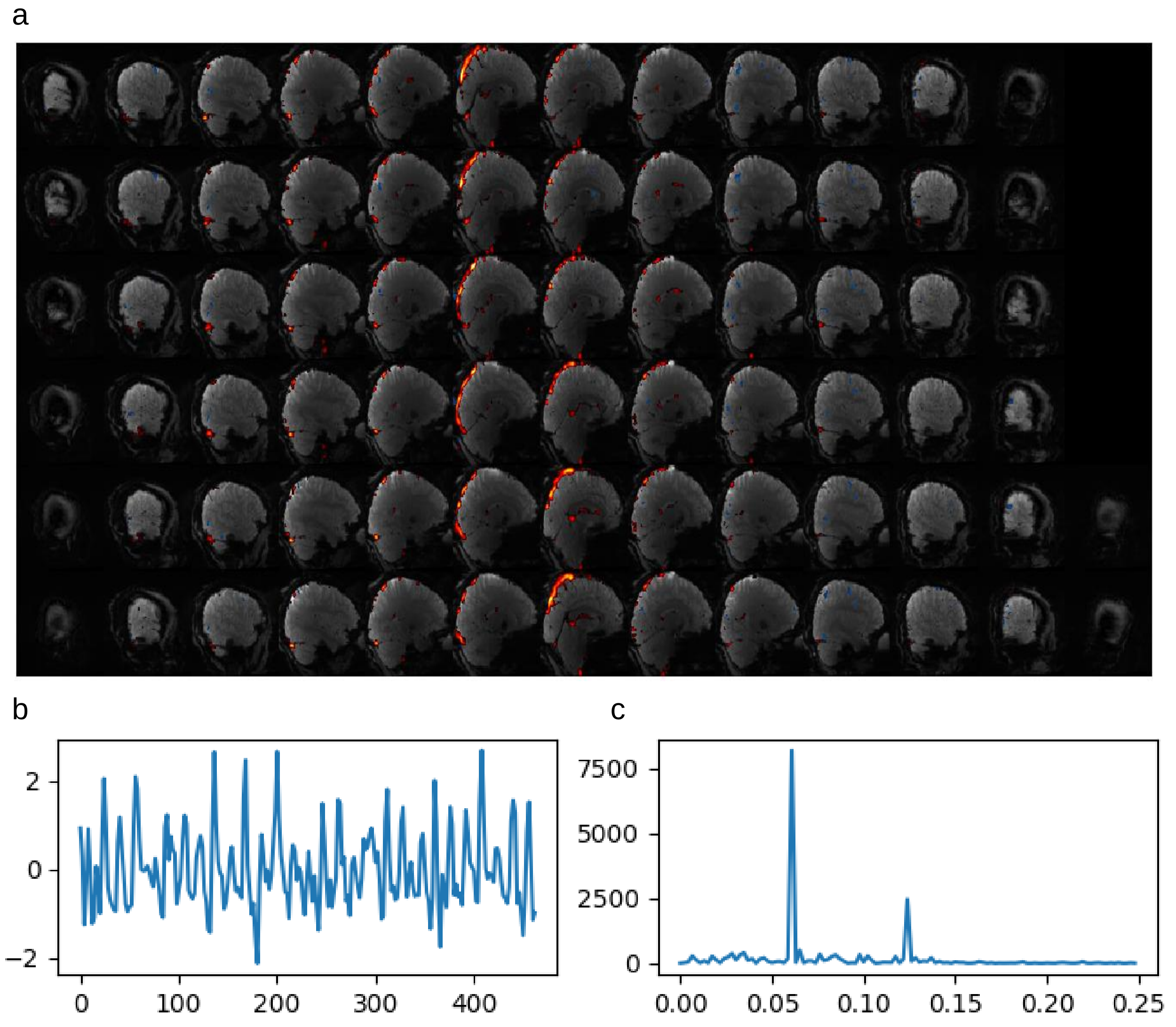

Supplementary Figure 8. A-IC example, Sagittal sinus (sub-31, run-1, IC-031). The spatial map of this category showed in sagittal sinus **(a)**. The time series of this category showed regular oscillatory patterns, without sudden jumps or gradual change **(b)**. The spectral power of this category lied in predominantly in low frequencies (i.e., < 0.1Hz) **(c)**.

Supplementary Figure 9. White matter

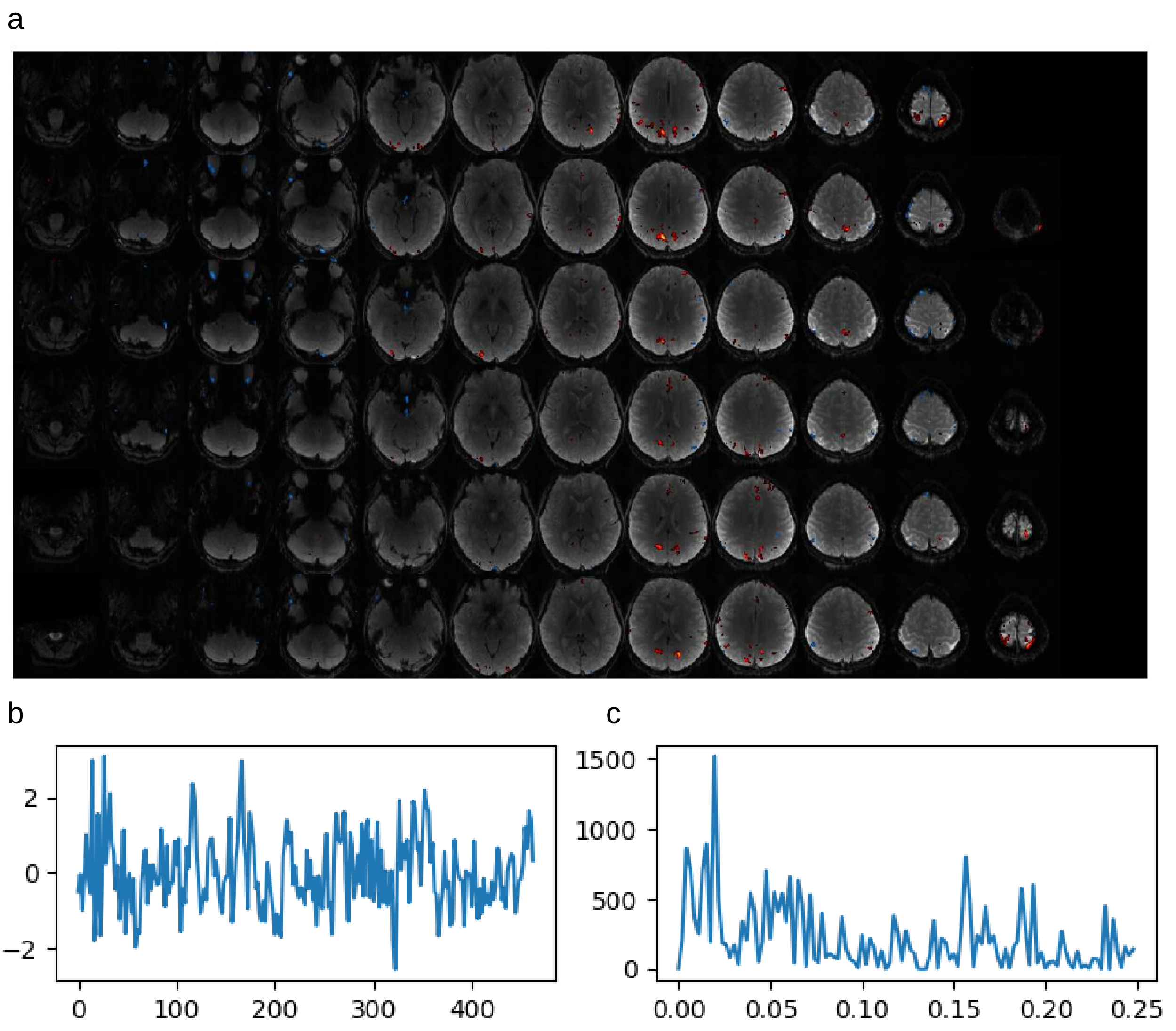

Supplementary Figure 9. A-IC example, White matter (sub-29, run-6, IC-093). The spatial map of this category showed in white matter **(a)**. The time series of this category showed regular oscillatory patterns, without sudden jumps or gradual change **(b)**. The spectral power of this category lied in broad bands **(c)**.

Supplementary Figure 10. MRI related noise

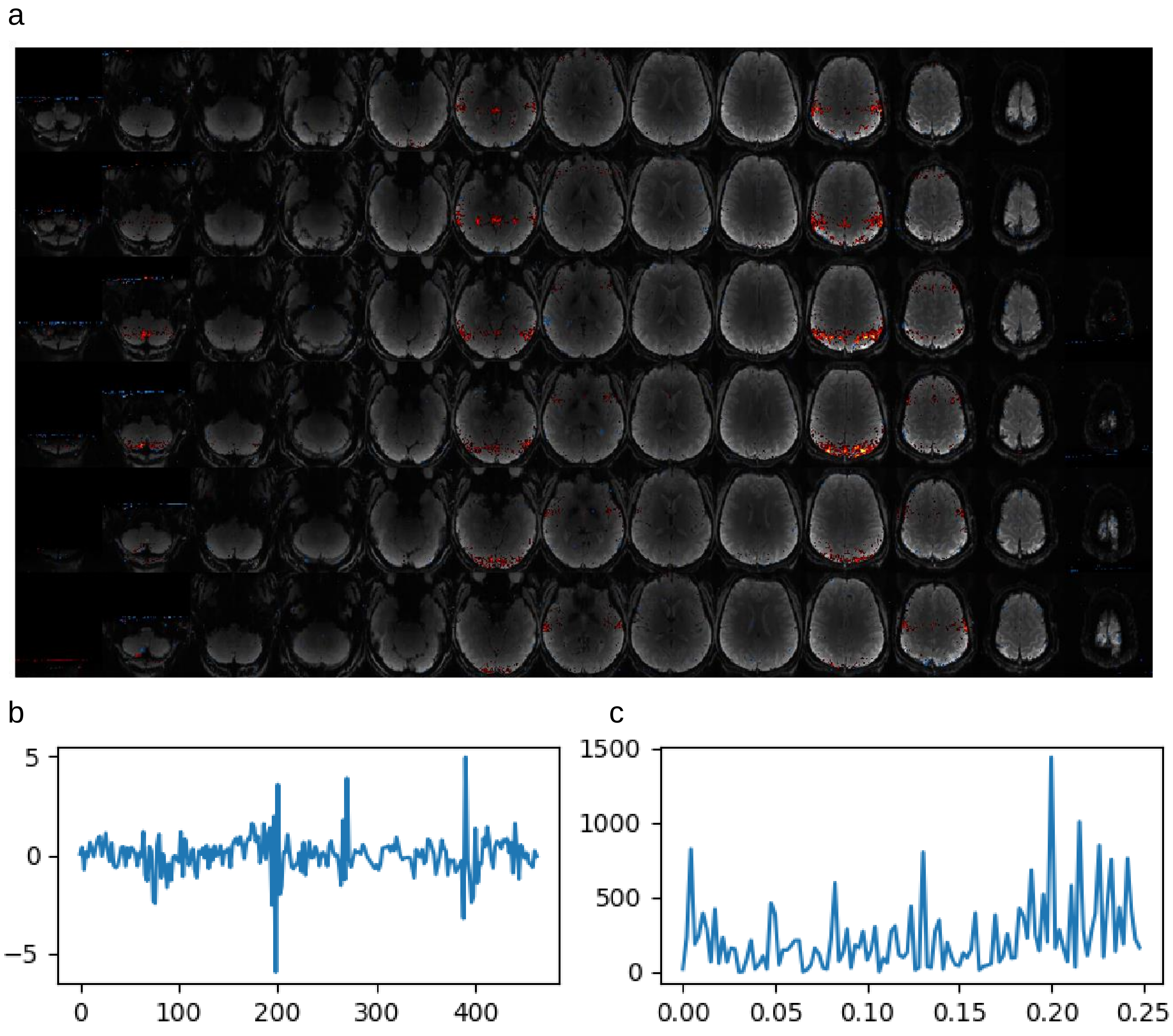

Supplementary Figure 10. A-IC example, MRI related noise (sub-57, run-1, IC-107). The spatial map of this category showed abrupt intensity changes in slice direction **(a)**. The time series of this category showed sudden jumps or oscillation patterns **(b)**. The spectral power of this category lied predominantly in high frequencies (i.e., > 0.1Hz) **(c)**.

Supplementary Figure 11. Unclassified noise

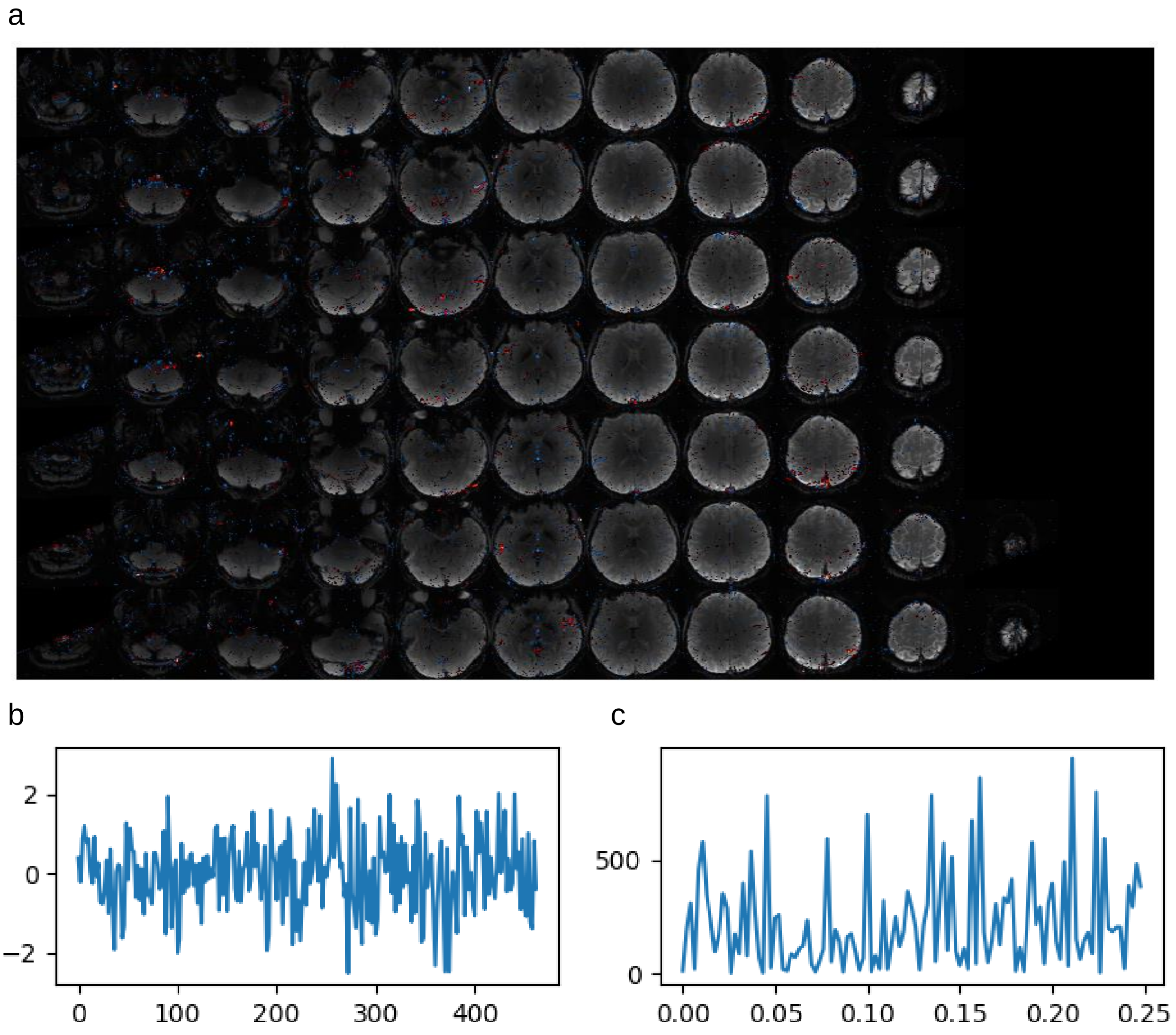

Supplementary Figure 11. A-IC example, Unclassified noise (sub-49, run-2, IC-050). The spatial map of this category showed a mixture of multiple kind of artifacts **(a)**. The time series of this category showed oscillation patterns **(b)**. The spectral power of this category lied in broad bands **(c)**.
